# The Expression of Functional Vpx during Pathogenic SIVmac Infections of Rhesus Macaques Suppresses SAMHD1 in CD4^+^ Memory T Cells

**DOI:** 10.1371/journal.ppat.1004928

**Published:** 2015-05-21

**Authors:** Masashi Shingai, Sarah Welbourn, Jason M. Brenchley, Priyamvada Acharya, Eri Miyagi, Ronald J. Plishka, Alicia Buckler-White, Peter D. Kwong, Yoshiaki Nishimura, Klaus Strebel, Malcolm A. Martin

**Affiliations:** 1 Laboratory of Molecular Microbiology, National Institute of Allergy and Infectious Diseases, National Institutes of Health, Bethesda, Maryland, United States of America; 2 Vaccine Research Center, National Institute of Allergy and Infectious Diseases, National Institutes of Health, Bethesda, Maryland, United States of America; University of Wisconsin, UNITED STATES

## Abstract

For nearly 20 years, the principal biological function of the HIV-2/SIV Vpx gene has been thought to be required for optimal virus replication in myeloid cells. Mechanistically, this Vpx activity was recently reported to involve the degradation of Sterile Alpha Motif and HD domain-containing protein 1 (SAMHD1) in this cell lineage. Here we show that when macaques were inoculated with either the T cell tropic SIVmac239 or the macrophage tropic SIVmac316 carrying a Vpx point mutation that abrogates the recruitment of DCAF1 and the ensuing degradation of endogenous SAMHD1 in cultured CD4^+^ T cells, virus acquisition, progeny virion production in memory CD4^+^ T cells during acute infection, and the maintenance of set-point viremia were greatly attenuated. Revertant viruses emerging in two animals exhibited an augmented replication phenotype in memory CD4^+^ T lymphocytes both *in vitro* and *in vivo*, which was associated with reduced levels of endogenous SAMHD1. These results indicate that a critical role of Vpx *in vivo* is to promote the degradation of SAMHD1 in memory CD4^+^ T lymphocytes, thereby generating high levels of plasma viremia and the induction of immunodeficiency.

## Author Summary

Primate lentiviruses, such as HIV and its SIV simian relative, encode accessory proteins that suppress cellular restriction factors interfering with efficient replication. One of these, designated Vpx, is produced in infected cells by HIV-2 and some SIV strains, which cause endemic infections in African monkeys. The primary function of Vpx has long been thought to facilitate infectivity in dendritic cells and macrophage by degrading the Sterile Alpha Motif and HD domain-containing protein 1 (SAMHD1), which restricts virus replication in these cells. Using SIVmac carrying a mutated Vpx gene with a single amino acid change that prevents it from binding to DCAF1 and subsequently mediating the degradation of SAMHD1, we show that virus infection of CD4^+^ T lymphocytes is markedly compromised both *in vitro* and *in vivo*. The SIV Vpx mutant is severely attenuated in establishing new infections in inoculated rhesus monkeys, in producing high levels of virus progeny, in degrading SAMHD1 in memory CD4^+^ T cell in infected animals, and in inducing symptomatic disease. Thus, although once considered to be only critical for optimal replication in macrophage based on earlier studies performed with cultured cells, the SIV Vpx protein is functionally important *in vivo* for establishing the primary infection in rhesus macaques, sustaining high levels of virus replication in CD4^+^ T lymphocytes, and promoting the onset of symptomatic immunodeficiency.

## Introduction

The Vpx accessory protein is encoded by HIV-2, related SIVsm strains, SIVmnd, and SIVrcm [1–4]. Vpx has been reported to antagonize restriction imposed by SAMHD1 in cultured myeloid lineage (dendritic cells, monocytes, and macrophages) and quiescent CD4^+^ T cells [5–8]. Early studies also showed that SIVmac239, carrying *vpx* gene deletions, exhibited an attenuated replication phenotype in inoculated macaques [9,10]. It is presently unclear whether compromised infection of myeloid lineage cells *in vivo* is responsible for this phenotype or if endogenous SAMHD1 must also be suppressed in memory CD4^+^ T lymphocytes, the cell lineage that sustains high levels of set-point viremia attending pathogenic infection.

Although the HIV-1 genome does not encode Vpx, most studies assessing Vpx degradation of SAMHD1 during virus infections have utilized pseudotyped HIV-1 constructs, in combination with SIV VLPs expressing Vpx, in single-cycle replication assays. Only a single study has utilized replication-competent HIV-1 to monitor Vpx-mediated suppression of SAMHD1 during an *in vitro* infection. In that experiment, SAMHD1 was reported to block virus infection in resting human CD4^+^ T lymphocytes unless SIVmac239 Vpx was co-packaged into an HIV-1 expressing GFP construct [5]. However, even though SAMHD1 levels had been markedly depleted and HIV-1 directed GFP expression became detectable intracellularly in the presence of Vpx, no progeny virions were produced.

The relevance of these *in vitro* functional studies of Vpx to the induction of immunodeficiency during pathogenic infections of macaques with SIVsm strains, such as SIVmac, in which the *vpx* gene is an intrinsic and evolutionarily conserved element, is not clear. It has been suggested that the antiviral activity of endogenous SAMHD1 may be limited to non-cycling cell lineages such as terminally differentiated myeloid cell subsets or, more recently, quiescent CD4^+^ T lymphocytes. Non-cycling memory CD4^+^ T lymphocytes are, in fact, the principal targets of both HIV and SIV during the initial weeks of the acute *in vivo* infection. Prodigious numbers of resting memory CD4^+^ T cells become infected in lymphoid tissues and blood and large amounts of circulating progeny virions are produced during this phase of the infection [11–13]. Furthermore, the relatively low levels of set point viremia and slow disease progression previously reported in rhesus macaques inoculated with SIV Vpx deletion mutants [9,10] suggests that Vpx may also be functionally important in counteracting SAMHD1 in virus-producing CD4^+^ memory T lymphocytes during the later chronic phase of the *in vivo* infection.

Here we examine replication-competent SIV Vpx mutants, disabled in their capacity to degrade SAMHD1. We show that when macaques were inoculated with SIV carrying the Q76A Vpx point mutation, which specifically affects the interaction of Vpx with DCAF1 and the subsequent recruitment of SAMHD1 for degradation, virus acquisition, progeny virion production in memory CD4^+^ T lymphocytes during the acute infection, and the maintenance of set point viremia were greatly attenuated. Revertant viruses, which emerged in two infected animals, carried substitutions located in likely contact points of Vpx with the c-terminal domain of DCAF1. Thus our data indicate that contrary to the commonly held belief that the principal function of SIV Vpx is to facilitate virus replication in myeloid lineage cells, the need to degrade endogenous SAMHD1 during SIV infections of memory CD4^+^ T cells *in vivo* is critically important and drives the selection for Vpx revertant viruses, capable of mediating the degradation of SAMHD1 and generating high levels of plasma viremia.

## Results

### SIVmac Vpx mutants generate lower amounts of progeny virions during infections of cultured rhesus PBMC and MDM

**Fig 1:**
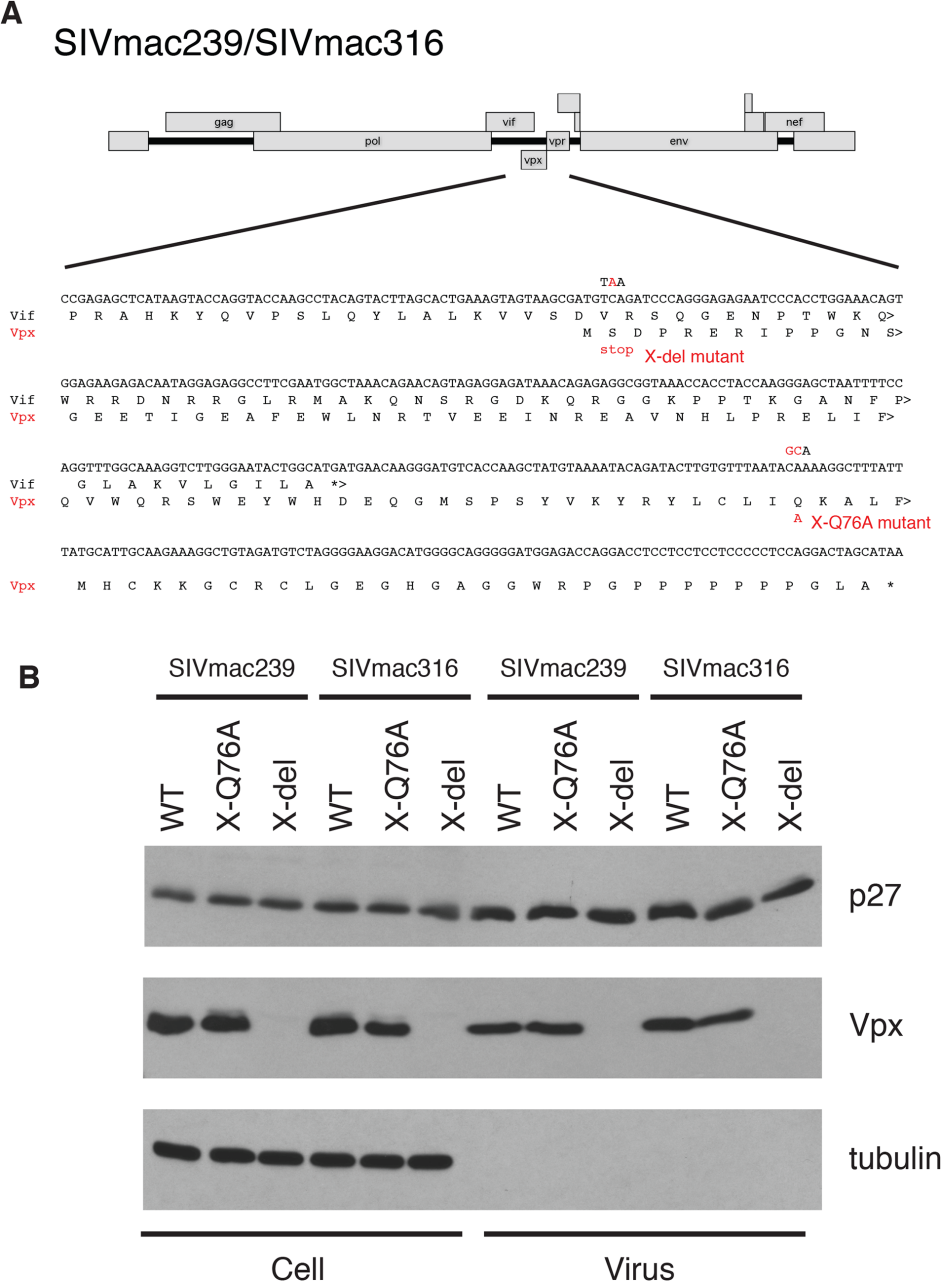
Construction of the SIVmac239 and SIVmac316 X-del and X-Q76A Vpx mutants. (A) The codon (TCA) for the second residue (serine) of SIV Vpx was changed to a stop codon (TAA) to generate the X-del Vpx mutant. This change does not alter the amino acid sequence of the overlapping Vif protein. In the X-Q76A Vpx mutant, amino acid 76 (glutamine) was changed to alanine by altering the CAA codon to GCA. (B) The intracellular expression of Vpx proteins and their incorporation into virions was evaluated by transfecting HeLa cells with WT and Vpx mutant full-length SIV plasmids. Forty-eight hours later, cell lysates or cell-free culture supernatants, pelleted through 20% sucrose, were evaluated by immunoblotting using anti-SIV plasma, anti-Vpx antibodies, and anti-tubulin antibodies.

Several earlier studies have reported that replication competent HIV-2 and SIVmac mutants, unable to express the Vpx protein, exhibit delayed infection kinetics and low levels of progeny virus production in cultured rhesus peripheral blood mononuclear cells (PBMC) [9,14,15]. To ascertain whether a Vpx point mutant, specifically defective in recruiting DCAF1 and subsequently degrading SAMHD1[7,16–18], might possess similar properties, the X-Q76A Vpx mutant, carrying a 2-nucleotide substitution, was constructed (Fig 1A). A second Vpx mutant (X-del), containing a TAA stop codon at residue 2 that prevents the synthesis of any SIV Vpx protein was also prepared (Fig 1A). Both Vpx mutations were introduced into molecular clones of wild type (WT) T cell tropic (SIVmac239) or WT macrophage tropic (SIVmac316) SIVs. Wild type and Vpx mutant SIV inocula were prepared by transfecting full-length infectious molecular clones into 293T cells as described in Methods. Vpx expression in HeLa cells and its incorporation into progeny virions released into the transfection supernatant medium were confirmed by immunoblotting (Fig 1B).

**Fig 2:**
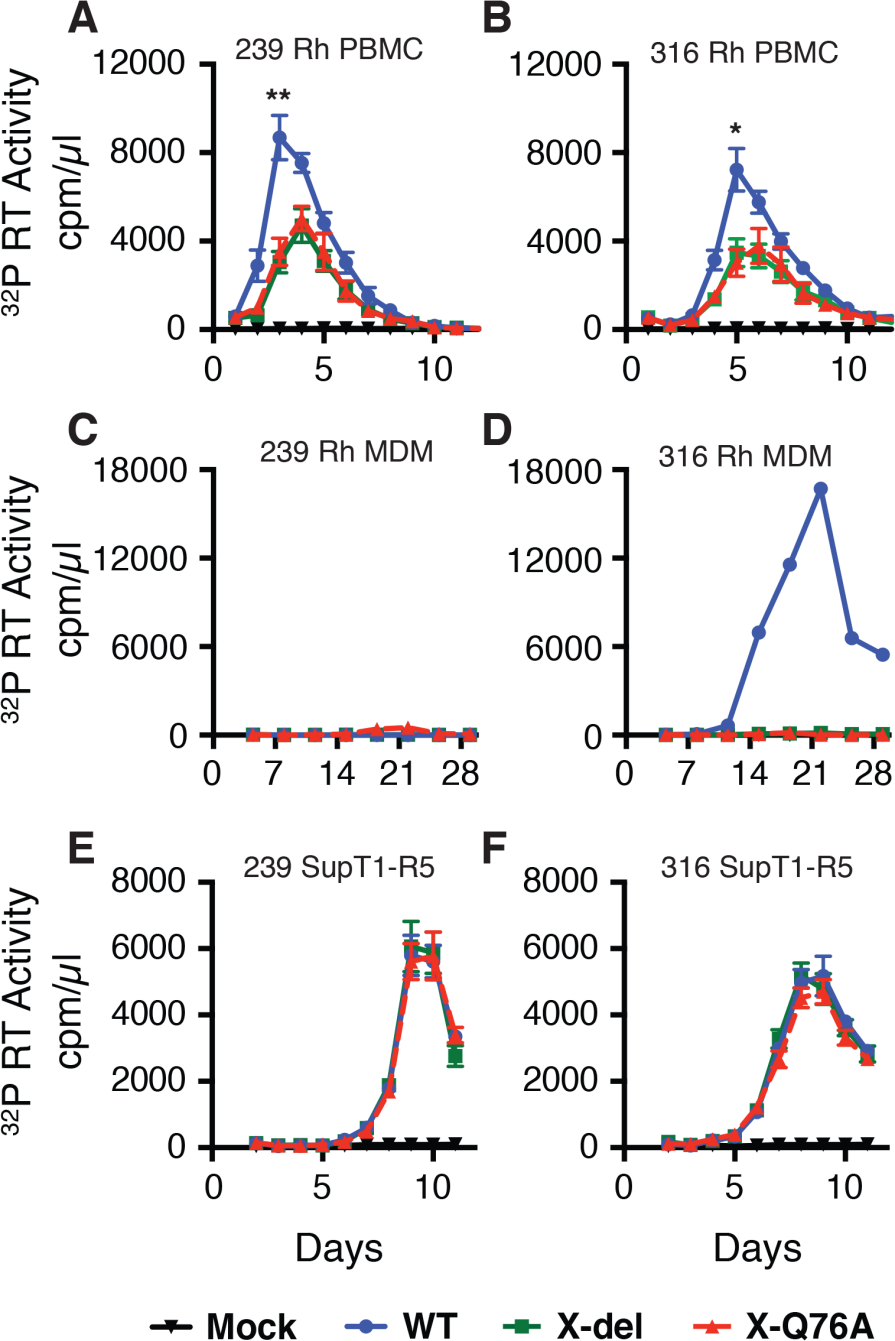
The replication of Vpx defective SIV mutants is attenuated in rhesus macaque mononuclear cells. ConA-activated rhesus PBMCs (A and B), rhesus MDM (C and D) and SupT1-R5 cells (E and F) were infected with WT and Vpx mutant SIV stocks produced in transfected 293T cells. The indicated cell cultures were infected with equivalent amounts of virus inocula, based on particle-associated ^32^P -RT activity (approximately 5 × 10^6^ cpm) and progeny virion production was monitored by measuring the RT activity released into the culture medium. The results in panels A and B, are shown as mean +/—s.e.m (n = 4). The parametric unpaired t test was performed using PRISM software. The significant p values (* p<0.05, ** p<0.01) for WT versus the X-del and X-Q76A Vpx mutants refer to the single time points at peak virus production. A representative result from at least two experiments is shown in panels C and D.

Compared to the WT viruses, both types of SIVmac239 or SIVmac316 Vpx mutants generated reduced amounts of progeny virions during infections of cultured Concanavalin A (ConA) stimulated rhesus PBMC (Fig 2A and 2B). The defective replication phenotype of the Vpx mutants was more profound during infections of rhesus monocyte derived macrophage (MDM) infections (Fig 2C and 2D). In contrast to the robust replication of the WT macrophage tropic SIVmac316 in rhesus MDM, replication of both the SIVmac316 Vpx deletion mutant and the SIVmac316 Vpx point mutant was blocked in these cells. As expected, neither the WT nor the Vpx mutants of the T cell tropic SIVmac239 were able to infect rhesus MDM (Fig 2C). The SIVmac239 and SIVma316 X-Q76A Vpx point mutants both exhibited replication kinetics in human SupT1-R5 cells indistinguishable from corresponding WT viruses (Fig 2E and 2F), consistent with results reporting that SupT1-R5 cells do not express the SAMHD1 protein [8], and confirming the absence of any inherent replication defects in these mutant viruses.

### SIVmac Vpx mediates the degradation of endogenous SAMHD1 during productive infections of cultured rhesus CD4^+^ T cells

**Fig 3:**
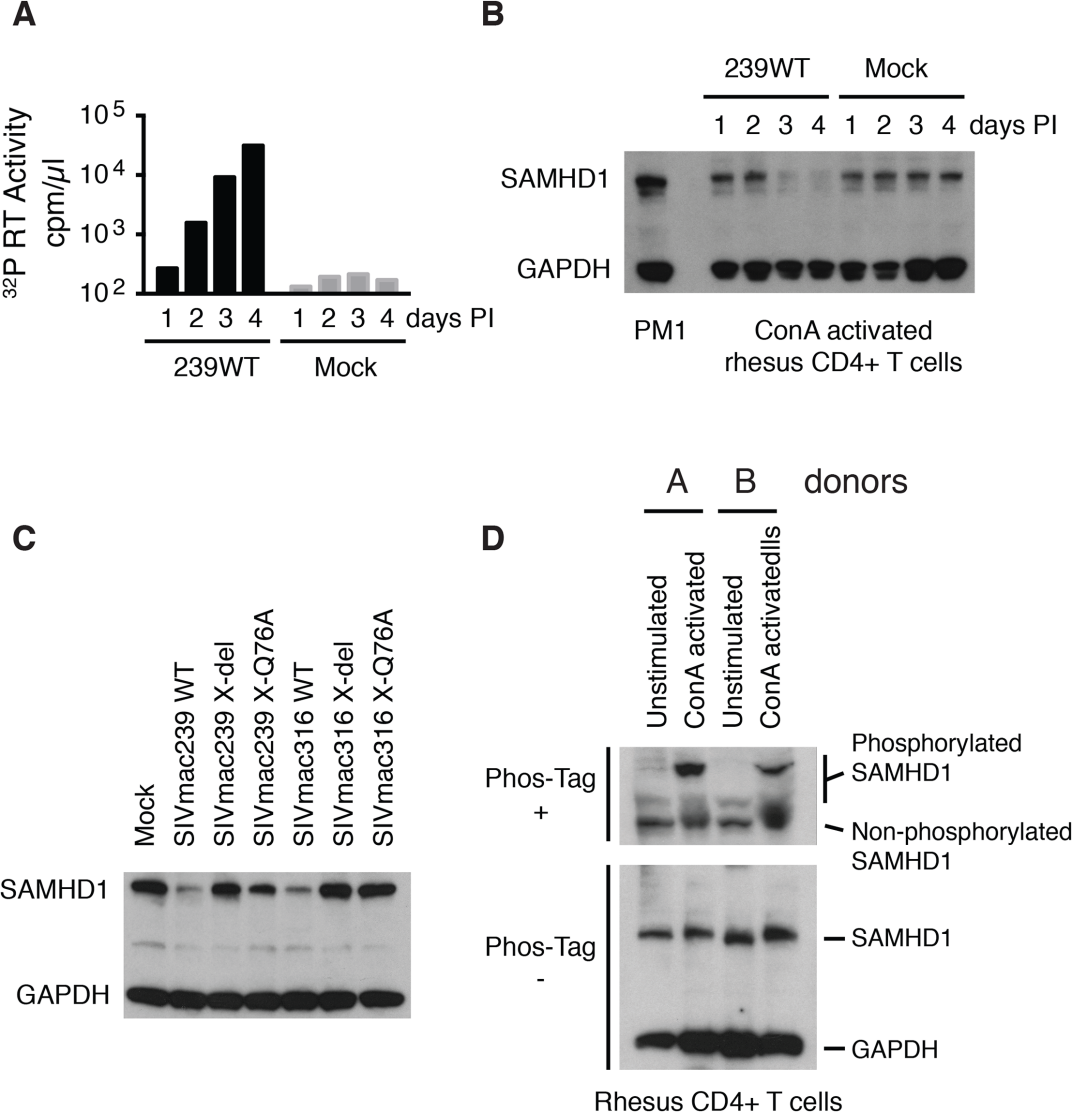
The presence of wild type Vpx correlates with reduced levels of endogenous SAMHD1 in ConA-activated macaque CD4^+^ T lymphocytes. CD8^+^ T cell-depleted rhesus PBMCs were activated with ConA for 24 h, cultured with IL-2 containing medium for 48 h, negatively selected for CD4^+^ T cells, and infected with SIVmac239 (MOI = 0.2). Cells and supernatant media were collected daily from the infected or mock-infected “enriched” CD4^+^ T lymphocyte cultures. (A) Progeny virions released into the medium were detected by ^32^P RT assay. (B) Levels of endogenous SAMHD1 present in SIVmac239 or mock infected cell extracts from 3 x 10^5^ cells were determined by immunoblotting following polyacrylamide gel electrophoresis using anti-SAMHD1 antibody. GAPDH was used as a loading control. (C) Individual CD4^+^ T lymphocyte cultures were infected with WT SIVmac239, WT SIVmac316, or corresponding Vpx derived mutants (MOI = 0.2). Based on the results shown in Panels A and B, cells were harvested on day 3 PI and levels of endogenous SAMHD1 present in virus or mock infected cell extracts were determined by immunoblotting. (D) SAMHD1 phosphorylation status in macaque CD4^+^ T lymphocytes was determined by electrophoresis in acrylamide gels with/without Phos-Tag and analyzed by immunoblotting. Whole-cell extracts (from 3 × 10^5^ cells) of non-activated and ConA-activated CD4^+^ T lymphocytes from two uninfected animals were separated on acrylamide gels with/without Phos-Tag and analyzed by immunoblotting using anti-SAMHD1 and GAPDH antibodies.

As noted earlier, a majority of previous studies evaluating virion-associated Vpx-mediated degradation of SAMHD1 have been conducted in non-lymphoid cells using pseudotyped virus preparations capable of only single cycles of replication. To determine whether Vpx-mediated degradation of endogenous SAMHD1 occurred during the course of spreading infections of replication-competent virus in CD4^+^ T lymphocytes, freshly collected, and negatively selected, Con-A stimulated rhesus macaque CD4^+^ T cells were infected with WT SIVmac239 at a multiplicity of infection (MOI) = 0.2. Cells and supernatant samples were collected daily and examined for levels of progeny virions released into the medium (^32^P-reverse transcriptase [RT] activity) and endogenous SAMHD1 (immunoblotting). Newly produced virus first became detectable on day 2 post infection (PI) and steadily increased on days 3 and 4 (Fig 3A). The levels of endogenous SAMHD1, present in the CD4^+^ T cells on days 1 and 2, markedly declined on days 3 and 4 PI when compared to an unrelated cellular protein (GAPDH) (Fig 3B). In an independent experiment, also assessing the status of endogenous SAMHD1, ConA-activated rhesus CD4^+^ T lymphocyte cultures were infected with WT SIVmac239, WT SIVmac316, or the two different and corresponding Vpx defective mutants, all at a MOI = 0.2. Based on the results of the experiment shown in Fig 3A and 3B, cells were collected on day 3 PI and lysates were examined by immunoblotting for levels of endogenous SAMHD1. As shown in Fig 3C, Con A-stimulated rhesus CD4^+^ T cells, infected with both of the WT SIVmac viruses, contained reduced levels of endogenous SAMHD1 compared to levels in mock infected cells or in cells infected with the corresponding Vpx mutant viruses. Together, these results demonstrate that SIV Vpx mutants bearing the Q76A point mutation, which specifically blocks the recruitment of DCAF1, are defective in degrading endogenous SAMHD1 (Fig 3) and are attenuated during spreading infections in cultured activated PBMC (Fig 2) compared to their WT counterparts.

It has recently been reported that phosphorylation of human SAMHD1 at the threonine 592 residue (Thr592) greatly reduces its capacity to restrict HIV-1 replication, but does not affect its dNTPase activity [19–21]. To first verify that the Thr592 residue was actually present in rhesus SAMHD1, mRNAs were prepared from a mixture of PBMC samples collected from six monkeys, amplified by RT-PCR, and individual amplicons were analyzed by nucleotide sequencing. Although rhesus and human SAMHD1 proteins differed at 38 of 626 amino acid positions, the Thr592 residue was present in both proteins (S1 Fig). It was therefore of interest to ascertain the phosphorylation status of SAMHD1 in primary rhesus mononuclear cells, particularly in the Con A-activated rhesus PBMC in which SIV production was shown modestly reduced in the absence of Vpx (Fig 2A and 2B). Cell lysates from either non-activated or ConA activated CD4^+^ T cells, from two different macaques, were subjected to electrophoresis in Phos-tag Acrylamide and immunoblotting using the anti-SAMHD1 antibody. As shown in Fig 3D, a significant fraction of SAMHD1 was phosphorylated in ConA activated rhesus CD4^+^ T cells, whereas in unstimulated CD4^+^ T cells, the majority of SAMHD1 was unphosphorylated. Taken together, the increased levels of phosphorylated nonrestrictive SAMHD1 in the stimulated PBMC cultures may very well have minimized the differences between the infectivities of WTand the Vpx mutants measured in these cells.

### The expression of SIVmac Vpx correlates with reduced levels of endogenous SAMHD1 during productive infections of rhesus macaques

**Fig 4:**
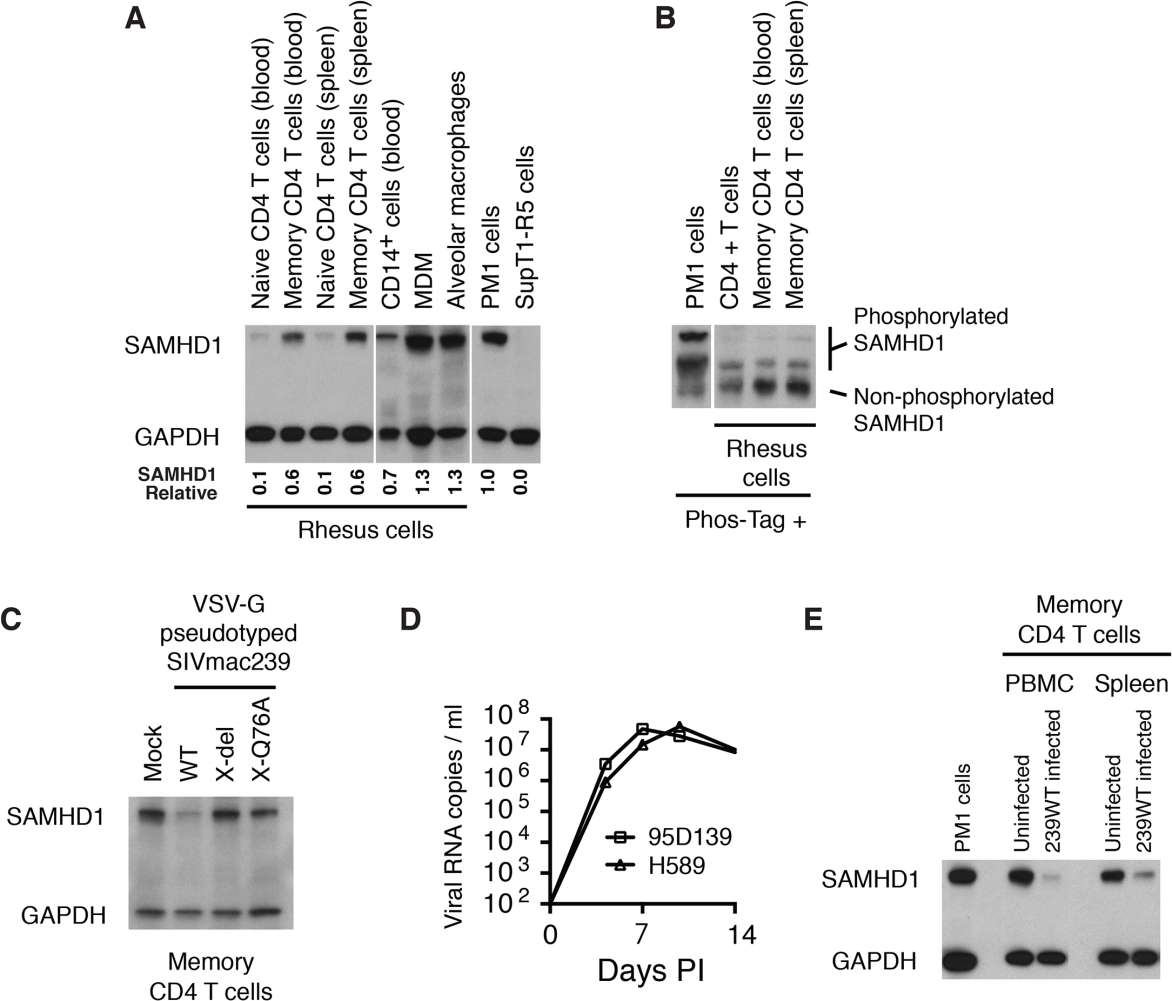
WT SIVmac239 degrades endogenous SAMHD1 in memory CD4^+^ T cells during the acute infection of rhesus macaques. (A) Levels of endogenous SAMHD1 expression were examined by immunoblotting rhesus macaque naïve and memory CD4^+^ T cells in blood and spleen, and myeloid lineages (CD14^+^ cells, MDM, and alveolar macrophages) or in two control human T cell leukemia lines (PM1 and SupT1-R5). Whole-cell extracts (from 3 × 10^5^ cells/lane) from each indicated source were separated on 10% acrylamide gels and stained using anti-SAMHD1 antibody. GAPDH was used as a loading control. The numbers below each lane indicate relative densitometric intensities of SAMHD1 bands relative to that of GAPDH and normalized to that present in PM1 cells. (B) SAMHD1 phosphorylation status in macaque memory CD4^+^ T lymphocytes was determined by electrophoresis in acrylamide gels with Phos-Tag and analyzed by immunoblotting. Whole-cell extracts (3 × 10^5^ cells) from PM1 cells, freshly collected rhesus CD4^+^ T cells or sorted rhesus memory CD4^+^ T cells from blood or spleen were separated on acrylamide gels with Phos-Tag and analyzed by immunoblotting using anti-SAMHD1 antibody. (C) Levels of SAMHD1 in sorted rhesus memory CD4^+^ T cells infected with VSV-G pseudotyped WT SIVmac239, SIVmac239 X-del or SIVmac239 X-Q76A were assessed by immunoblotting using anti-SAMHD1 antibody. (D) Acute phase viremia in two macaques inoculated intravenously with 10,000 TCID_50_ of SIVmac239. (E) Memory CD4^+^ T cells were prepared by cell sorting from PBMC or spleen from an uninfected or an infected rhesus macaque at day 9 PI following IV inoculation with 10,000 TCID_50_ of SIVmac239. The plasma viral load on day 9 post infection in the macaque inoculated with WT SIVmac239 was 7.8 x 10^7^ RNA copies/ml; viral RNA in the uninfected animals were below levels of detection (< 100 RNA copies/ml). Whole-cell extracts from 3 x 10^5^ sorted memory CD4^+^ T cells from each source or from PM1 cells were analyzed by immunoblotting using anti-SAMHD1 and anti-GAPDH antibodies.

*In vivo*, the principal target of both HIV-1 and SIVmac are memory CD4^+^ T lymphocytes [11,22,23]. The levels of SAMHD1 in unstimulated rhesus macaque memory CD4^+^ T cells, freshly collected from either blood or spleen, were examined by immunoblotting using anti-human SAMHD1 antibody. As shown in Fig 4A, higher levels of SAMHD1 were detected in memory CD4^+^ T lymphocytes than those measured in naïve CD4^+^ T cells from the same two sources. Among rhesus myeloid lineages, circulating CD14^+^ monocytes expressed levels of SAMHD1 similar to those present in memory CD4^+^ T cells, whereas much higher concentrations of SAMHD1 were detected in macaque alveolar macrophage, collected by bronchoalveolar lavage, and rhesus MDM derived *in vitro*. Cell lysates from the same preparations of freshly collected unstimulated rhesus macaque memory CD4^+^ T cells from blood or spleen were subjected to electrophoresis in Phos-tag Acrylamide and immunoblotting using the anti-SAMHD1 antibody. As shown in Fig 4B, most of the SAMHD1 in memory CD4^+^ T cells was unphosphorylated. The relatively high levels of expression of unphosphorylated endogenous SAMHD1 measured in memory CD4^+^ T lymphocytes suggested that SAMHD1 might significantly restrict viral infections *in vivo*, reduce virus production systemically, and affect disease progression if not adequately suppressed by Vpx.

To specifically examine whether Vpx can degrade endogenous SAMHD1 in rhesus memory CD4^+^ T cells, a large preparation of this subset was purified by FACS and infected with VSV-G pseudotyped WT SIVmac239 or its X-del or X-Q76Aderivatives; SAMHD1 expression was evaluated 24 h later by immunoblotting. As shown in Fig 4C, the levels of SAMHD1 in WT SIVmac239 infected memory CD4 T cells were markedly reduced compared to that in cells infected with the Vpx mutants.

More importantly, to directly ascertain whether Vpx also degrades endogenous SAMHD1 in memory CD4^+^ T cells *in vivo*, we next determined if SAMHD1 degradation could be detected in memory CD4^+^ T lymphocytes, collected from SIVmac239 infected macaques. During the first weeks of the SIV acute infection, CD4^+^ memory T lymphocytes are massively infected systemically [11]. For example, on day 10 post inoculation, levels of cell-associated SIV DNA were reported to be in the range of 1 x 10^5^ copies/10^5^ memory CD4^+^ T cells in PBMC, inguinal and mesenteric lymph nodes, and jejunum mucosa. The establishment of such a prodigious *in vivo* infection provided a window of opportunity to directly examine the status of endogenous SAMHD1 in memory CD4^+^ T cells during the acute infection. The infection kinetics of SIVmac239 in two previously described rhesus monkeys (95D132 and H589) [24], inoculated intravenously (IV) with 1 × 10^4^ TCID_50_ of SIVmac239 is shown in Fig 4D. Based on this result, a macaque was inoculated IV with 1 × 10^4^ TCID_50_ of WT SIVmac239, sacrificed on day 9 PI, and memory CD4^+^ T cells from PBMC and spleen were collected by flow cytometric sorting. Memory CD4^+^ T lymphocytes were similarly prepared from the PBMC and spleen of an uninfected monkey. Immunoblotting revealed markedly reduced levels of SAMHD1 in memory CD4^+^ T cells purified from PBMC and spleen in the day 9 infected animal compared to those present in similar cells from the uninfected monkey (Fig 4E). This result demonstrates that levels of endogenous SAMHD1 in memory CD4^+^ T lymphocytes are greatly diminished at the time of peak virus production *in vivo*, presumably reflecting SIVmac239 LTR-directed expression of Vpx.

### Virus acquisition is impaired and levels of set-point viremia are suppressed in macaques inoculated with SIV Q76A Vpx mutants

**Fig 5:**
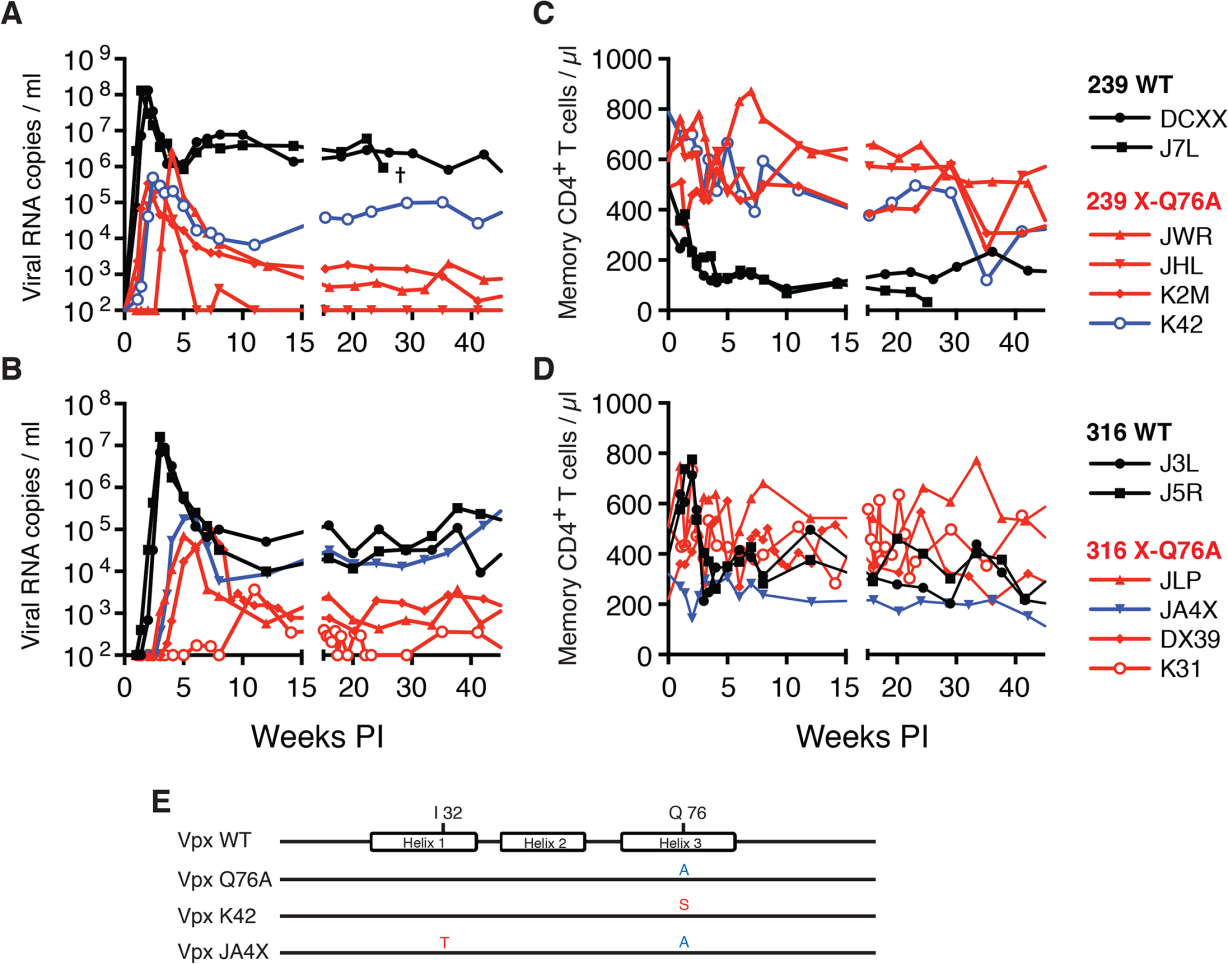
SIVs deficient in degrading SAMHD1 in memory CD4^+^ T cells exhibit an attenuated replication phenotype in inoculated rhesus macaques. Rhesus macaques were inoculated intrarectally with 1 x 10^4^ TCID_50_ of SIVmac239 WT or SIVmac239 X-Q76A derivatives (A and C) or 1 x 10^3^ TCID_50_ of SIVmac316 WT or the SIVmac316X-Q76A derivatives (B and D). The infectious virus titers in the inocula were determined by end-point dilution using SAMHD1 negative SupT1-R5 cells to avoid suppressive effects of SAMHD1 restriction. Plasma viral copies/ml are shown in panel A and C. Memory CD4+ T cell counts/μl are shown in panel C and D. Black curves: WT virus; blue curves: putative revertant Vpx mutants; red curves: non-revertant Vpx mutants. (E) Amino acid substitutions present in the starting Q76A Vpx mutant virus or in the putative revertant virus populations present in the plasmas of macaques K42 and JAX4 at week 35 PI, based on SGA (see Fig 6) are shown. The locations of the three helical domains of SIVmac Vpx are indicated.

An extensive literature exists reporting that SIVmac and HIV-2 Vpx is a major facilitator of virus replication in cultures of terminally differentiated myeloid cells [10,25–27]. In an attempt to translate this particular cell-dependent Vpx function to the organismal level, an experiment was designed to direct WT and Vpx-deficient viruses to at least one myeloid lineage cell type (*viz*. dendritic cells) by using a mucosal rather than an IV route of virus inoculation. Accordingly, two macaques (DCXX and J7L) were challenged intrarectally (IR) with 1 × 10^3^ TCID_50_ of WT SIVmac239 and two macaques (J3L and J5R) were inoculated by the same route with 1 × 10^3^ TCID_50_ of WT SIVmac316. Virus acquisition only occurred in the two WT SIVmac316 challenged monkeys (J3L and J5R) (Fig 5A and 5B). After waiting 6 additional weeks, the same two macaques (DCXX and J7L), which had previously been inoculated with 1 x 10^3^ TCID_50_ of WT SIVmac239, were re-inoculated intrarectally with 1 x 10^4^ TCID_50_ of SIVmac239. Both monkeys rapidly became infected and generated high levels of peak plasma viremia (Fig 5A). Based on these results, we elected to inoculate four additional macaques intrarectally with 1 x 10^4^ TCID_50_ of the SIVmac239 X-Q76A Vpx mutants and four other animals with 1 x 10^3^ TCID_50_ of the SIVmac316 X-Q76A Vpx mutant to assess the infectivities of the Vpx point mutant viruses *in vivo*.

**Fig 6:**
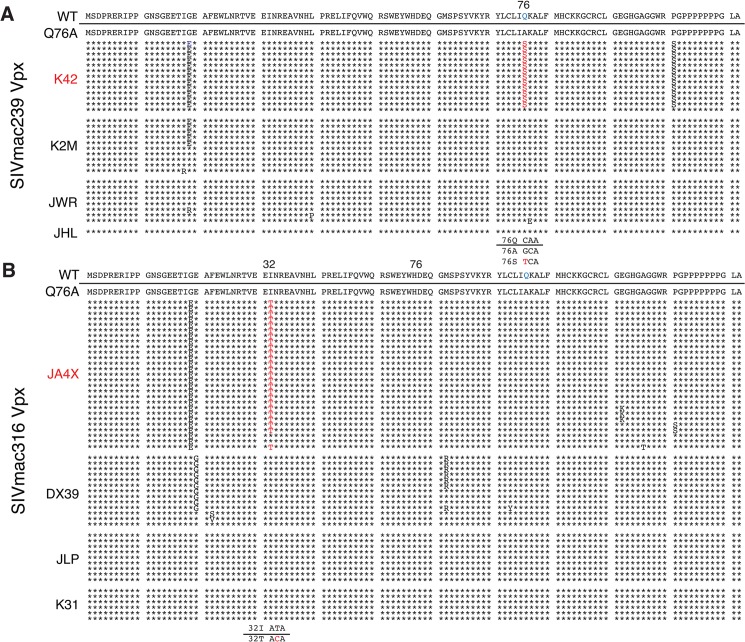
Alignment of Vpx amino acid sequences amplified from the plasma of monkeys inoculated with SIV Vpx X-Q76A mutants. SGA plus sequencing was used to generate *vpx* gene sequences present in the plasma of (A) SIVmac239 X-Q76A infected animals (K42, K2M, JWR, and JHL) and (B) SIVmac316 X-Q76A infected animals (JA4X, DX39, JLP, and K31) at week 35 PI. The sequence of WT SIVmac Vpx is shown at the top and the animal identifications are indicated on the left. Amino acids highlighted in red represent changes conferring putative revertant phenotypic changes. Amino acid substitutions in black are not thought to contribute to revertant phenotype because when present alone and in the absence of a change in the starting Q76A Vpx mutation (*e*.*g*. in the K2M virus), they fail to restore WT properties. The nucleotide changes corresponding to the putative revertant amino acid substitutions at Vpx residue 76 or residue 32 are shown at the bottom of each panel.

Both macaques inoculated with WT SIVmac239 generated peak plasma viremia levels of 1.3 × 10^8^ RNA copies/ml between days 10 and 14 PI, and developed viral set points of 2.9 x 10^6^ and 6.0 x 10^6^ RNA copies/ml, respectively (Table 1). The four monkeys (JWR, JHL, K2M, and K42) inoculated by the IR route with the SIVmac239 Vpx Q76A point mutant all exhibited attenuated replication phenotypes (Fig 5A and Table 1). In three of these animals (JWR, K2M, and K42), peak plasma viral loads ranged from 3.40 × 10^5^ to 2.66 × 10^6^ RNA copies/ml, 50 to 400-fold lower levels than those measured in WT SIVmac239 infected macaques. In two of these monkeys, the time of peak virus production was delayed to day 17 or day 28 PI. One of the animals (JHL) required 3 successive IR inoculations of the SIVmac239 Vpx Q76A mutant, spaced 6 weeks apart, to establish the SIV infection; a relatively low peak plasma viremia (2.25 x 10^5^ RNA copies/ml) in this macaque was delayed until day 21 post challenge. It should also be noted that the viral set points in three of the animals inoculated with the SIVmac239 Vpx point mutant were markedly reduced compared to monkeys infected with WT virus (Fig 5A), falling below the level of detection in one macaque (JHL) by week 12 PI. As shown in Fig 5C, memory CD4^+^ T cells rapidly declined in the two monkeys inoculated with WT SIVmac239 but did not change appreciably in animals infected with the Vpx mutants. The memory CD4^+^ T cell subset, as a percentage of total CD4^+^ T cells in these two infected animals, rapidly declined compared to levels in the 4 macaques inoculated with the Vpx mutants (S2A Fig).

**Table 1:** Infection of rhesus macaques with WT SIVmac and Q76A Vpx SIVmac mutants.

Animal ID	Virus Strain	Inoculations	Peak Virus Load (RNA Copies/ml)	Peak Day	Set-Point Virus Load (RNA copies/ml)
**DCXX**	239 WT	1	1.32 x 10^8^	14	2.90 x 10^6^
**J7L**	239 WT	1	1.30 x 10^8^	10	6.04 x 10^6^
**JWR**	239 X-Q76A	1	2.66 x 10^6^	28	5.88 x 10^2^
**JHL**	239 X-Q76A	3	2.25 x 10^5^	21	< 100
**K2M**	239 X-Q76A	1	3.40 x 10^5^	14	1.47 x 10^3^
**K42**	239 X-Q76A	1	4.92 x 10^5^	17	5.55 x 10^4^
**J3L**	316 WT	1	9.00 x 10^6^	24	1.00 x 10^5^
**J5R**	316 WT	1	1.62 x 10^7^	21	2.99 x 10^4^
**JLP**	316 X-Q76A	1	7.05 x 10^4^	35	4.33 x 10^2^
**JA4X**	316 X-Q76A	2	2.00 x 10^5^	42	1.54 x 10^4^
**DX39**	316 X-Q76A	1	9.08 x 10^4^	52	2.00 x 10^3^
**K31**	316 X-Q76A	3	3.67 x 10^3^	77	< 100

A similar result was obtained with the WT macrophage tropic SIVmac316 and its Vpx Q76A mutant. As noted above, the two animals (J3L and J5R) inoculated with 1 × 10^3^ TCID_50_ of WT SIVmac316 became infected following a single challenge and developed levels of peak plasma viremia of 1.62 × 10^7^ and 9.00 × 10^6^ RNA copies/ml on days 21 and 24 PI, respectively (Fig 5B and Table 1). The set point levels of viremia (3.0 × 10^4^ and 1.0 × 10^5^ copies/ml) in these two monkeys were somewhat lower than those measured in the macaques inoculated with WT SIVmac239. When the SIVmac316 Vpx Q76A mutant was similarly evaluated in four animals, its replication properties were even more severely debilitated than the analogous SIVmac239 Vpx mutants (Fig 5B and Table 1). An infection was established in two of these four monkeys (JLP and DX39) following a single IR challenge, but the peak viral loads (7.05 ×10^4^ and 9.08 × 10^4^ RNA copies/ml) were 2 logs lower than that measured with WT SIVmac316 and were markedly delayed (until day 35 and day 52 post inoculation, respectively). In addition, the viral set points at weeks 22 to 24 in these two macaques were quite low (4.33 × 10^2^ and 2.00 × 10^3^ RNA copies/ml). Establishment of an SIVmac316 infection in the two remaining animals receiving the SIVmac316 Q76A Vpx mutant virus (JA4X and K31) required 2 and 3 successive inoculations, respectively, with a peak viral load reaching only 3.7 × 10^3^ RNA copes/ml at day 77 PI in the latter macaque (Fig 5B and Table 1). Taken together, these results indicate that the Q76A Vpx mutation is profoundly disabling *in vivo*, affecting SAMHD1 degradation, SIVmac acquisition, the production of progeny virions during the acute infection, and the maintenance of set-point viremia, all of which are replication functions that occur in memory CD4^+^ T cells. A modest reduction of memory CD4^+^ T lymphocytes occurred in the two monkeys inoculated with WT SIVmac316 but not in the animals infected with the Vpx mutants (Fig 5D). The memory CD4^+^ T cell subset in these two macaques, as a percentage of total CD4^+^ T cells, declined somewhat compared to levels measured in the monkeys inoculated with the Vpx mutants (S2B Fig).

### Vpx revertant viruses emerge in macaques inoculated with SIV Q76A Vpx mutants

During the chronic phase of their infections, one of the four recipients of the SIVmac239 Vpx mutant (macaque K42) and one of the four recipients of the SIVmac316 Vpx mutant (macaque JA4X) developed elevated set-point viremia levels that distinguished them from the six other monkeys inoculated with the Q76AVpx mutant (indicated by the blue curves in Fig 5A and 5B). In fact, the set-point virus load in animal JA4X was similar to those generated by the two recipients of WT SIVmac316 (Fig 5B). It should be noted that to minimize the emergence of revertant viruses during infections *in vivo*, a Q76A Vpx point mutant, containing a two nucleotide substitution, was purposely constructed.

The possible emergence of Vpx revertant viruses was initially investigated by performing single genome amplification (SGA) analyses of plasma samples collected at week 35 PI from all 8 monkeys inoculated with the SIVmac239 or the SIVmac316 Q76A Vpx point mutants (Fig 6). Nucleotide sequence analyses revealed that 13 of 14 *vpx* gene amplicons from the putative revertant virus circulating in macaque K42 had acquired an A76S (GCA to TCA) substitution at the site of the original Q76A mutation in the SIVmac239 Vpx mutant. In the case of the putative revertant virus recovered from monkey JA4X, all of the week 35 amplicons had retained the original Q76A mutation; however, 25 of 27 carried a “second site” I32T (ATT to ACA) change. Both putative Vpx revertant viruses also had acquired a G19E substitution (Fig 6). However, the latter was the only change present in some *vpx* gene amplicons from macaque K2M (Fig 6) and this single amino acid substitution failed to restore high levels of set-point viremia in the animal (see Fig 5A). The “second site” I32T Vpx substitution identified in virus circulating in macaque JA4X would be consistent with and support a recent genetic study reporting that Ile32 is a critical residue mediating Vpx and DCAF1 interactions [28]. The Vpx amino acid changes associated with revertant viruses isolated from macaques JA4X and K42 are located in helix 1 and helix 3, respectively, and are shown diagrammatically in Fig 5E.

**Fig 7:**
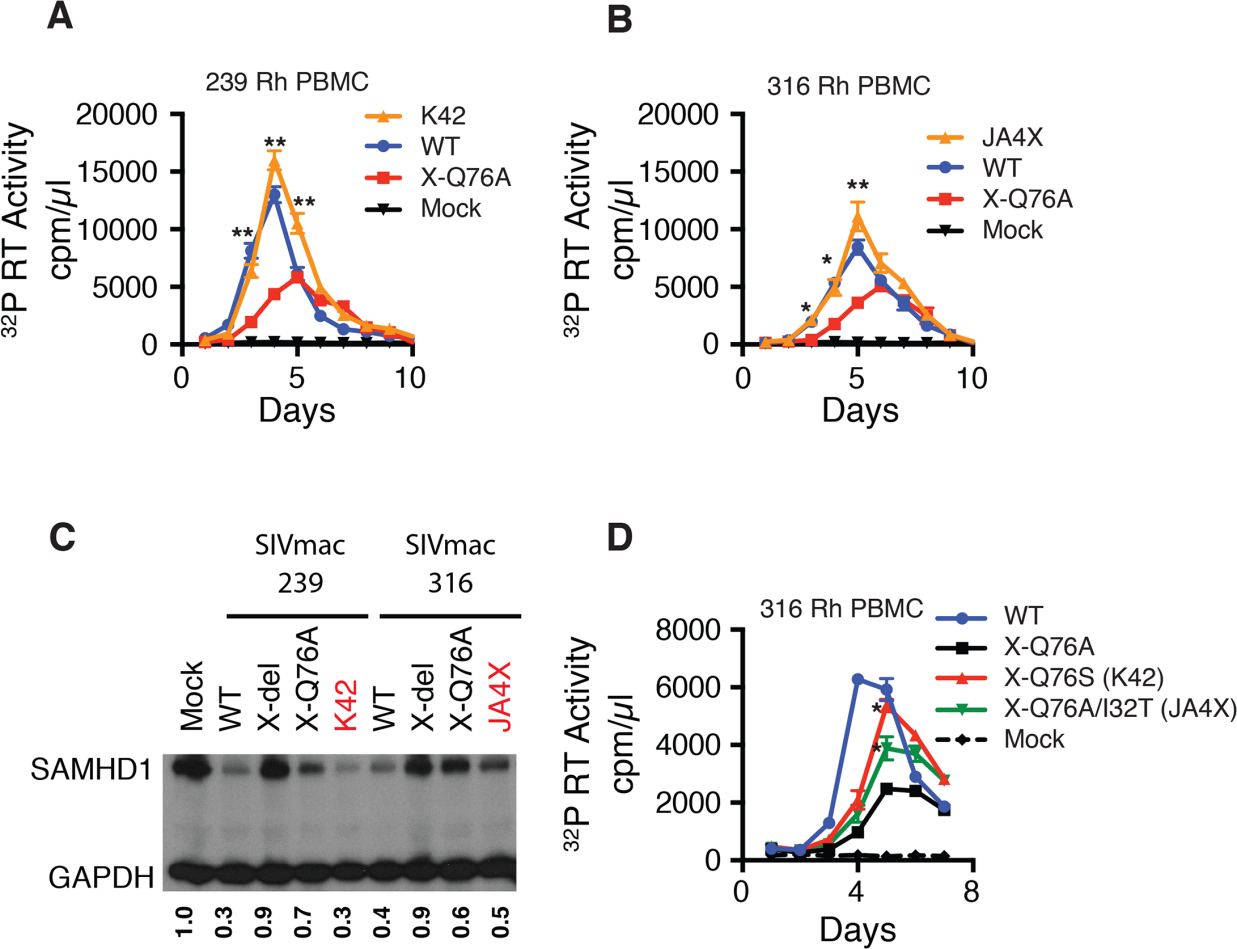
Analyses of Vpx revertant viruses emerging in macaques K42 and JA4X. ConA-activated rhesus PBMCs were infected at a MOI = 0.01 with (A) the WT SIVmac239, SIVmac239 X-Q76A Vpx mutant, or the virus recovered at week 40 PI from infected macaque K42 or (B) the WT SIVmac316, SIVmac316 X-Q76AVpx mutant, or the virus recovered at week 40 PI from infected macaque JA4X. Virus replication was monitored by measuring the particle-associated ^32^P RT activity released into culture supernatants. Results are shown as mean +/- s.e.m. The parametric unpaired t test was performed using PRISM software. The significant p values (* p<0.05, ** p<0.01) for putative revertant viruses compared to the starting X-Q76A Vpx mutant refer to the indicated time points during the infection. (C) ConA- activated enriched rhesus CD4^+^ T lymphocytes were infected with the indicated viruses at MOI = 0.2. Whole cell lysates were prepared on day 3 PI and levels of endogenous SAMHD1 present in virus or mock infected cell extracts from 3 x 10^5^ cells/lane were determined by immunoblotting as described in Fig 4C. The numbers below each lane indicate relative densitometric intensities of SAMHD1 bands relative to that of GAPDH and normalized to that present in Mock infected cells. (D) Rhesus PBMC were infected with the WT SIVmac316 or the molecularly cloned SIVmac316 derivatives containing the X-Q76A, Q76S, or the Q76A/I32T Vpx substitutions, using virus stocks prepared in transfected 293T cells. Cell cultures were infected with equivalent amounts of virus inocula, based on particle-associated RT activity (approximately 5 × 10^6 32^P cpm). Progeny virion production is shown as mean +/—s.e.m. The parametric unpaired t test was performed using PRISM software. The significant p values (* p<0.05) for SIVmac316 X-Q76S or SIVmac316 X-Q76A/I32T versus the starting SIVnac316 X-Q76A Vpx mutant are indicated. Representative results from at least two experiments are shown.

To ascertain whether revertants had, in fact, emerged in monkeys JA4X or K42, virus stocks were prepared from each animal by co-cultivating their PBMC, collected at week 40 PI, with SupT1-R5 cells. We then confirmed that these recovered swarm virus stocks (K42 and JA4X) had each retained their respective putative revertant mutation by SGA analysis: 10 of 10 amplicons from the K42 stock carried the Vpx A76S revertant change; 15 of 15 amplicons from the JA4X stock contained the original Vpx Q76A mutation as well as the I32T substitution (S3 Fig). The recovered viruses were then assayed for infectivity in rhesus macaque PBMC, side-by-side, with the corresponding starting Q76A Vpx mutant viruses. In contrast to the original Q76A Vpx mutants, which exhibited attenuated replication phenotypes, the viruses isolated from animals K42 or JA4X had robust infection kinetics in rhesus PBMC and released high levels of progeny virions (Fig 7A and 7B).

We next determined whether the capacity of the putative revertant viruses to degrade endogenous SAMHD1 was restored during productive *in vitro* infections. Rhesus CD4^+^ T lymphocyte cultures were prepared as described for Fig 3C and infected with the viruses recovered from monkeys K42 or JA4X, the parental WT SIVmac239, WT SIVmac316, and the two corresponding Q76A Vpx mutants. Infected cells were collected at day 3 PI and lysates were examined by immunoblotting for endogenous SAMHD1. As shown in Fig 7C, macaque CD4^+^ T lymphocytes infected with the two putative revertant viruses and both WT viruses contained lower levels of endogenous SAMHD1 compared to that present in cells infected with the starting Q76A Vpx mutant viruses. These results indicate that viruses recovered from macaques K42 and JA4X had re-acquired the capacity to degrade endogenous SAMHD1 as well as to release large amounts of progeny virions during spreading infections in Con-A activated rhesus CD4^+^ T cells.

The functional significance of the Vpx substitutions in revertant virus populations that had emerged was directly assessed by inserting the changes shown in Fig 5C into the genetic background of SIVmac316 and evaluating the replication phenotypes of the resultant constructs during infections of rhesus PBMC. As shown in Fig 7D, the X-Q76S revertant virus released nearly three-fold more progeny virions than the starting X-Q76A Vpx mutant in rhesus PBMC. The Q76A/I32T second-site revertant virus was less robust and generated intermediate amounts of virus. These results indicate that both revertant changes augmented virus replication in rhesus PBMC compared to the initial attenuated Q76A Vpx mutant.

### Structural analyses of the A76S and I32T Vpx revertant substitutions

**Fig 8:**
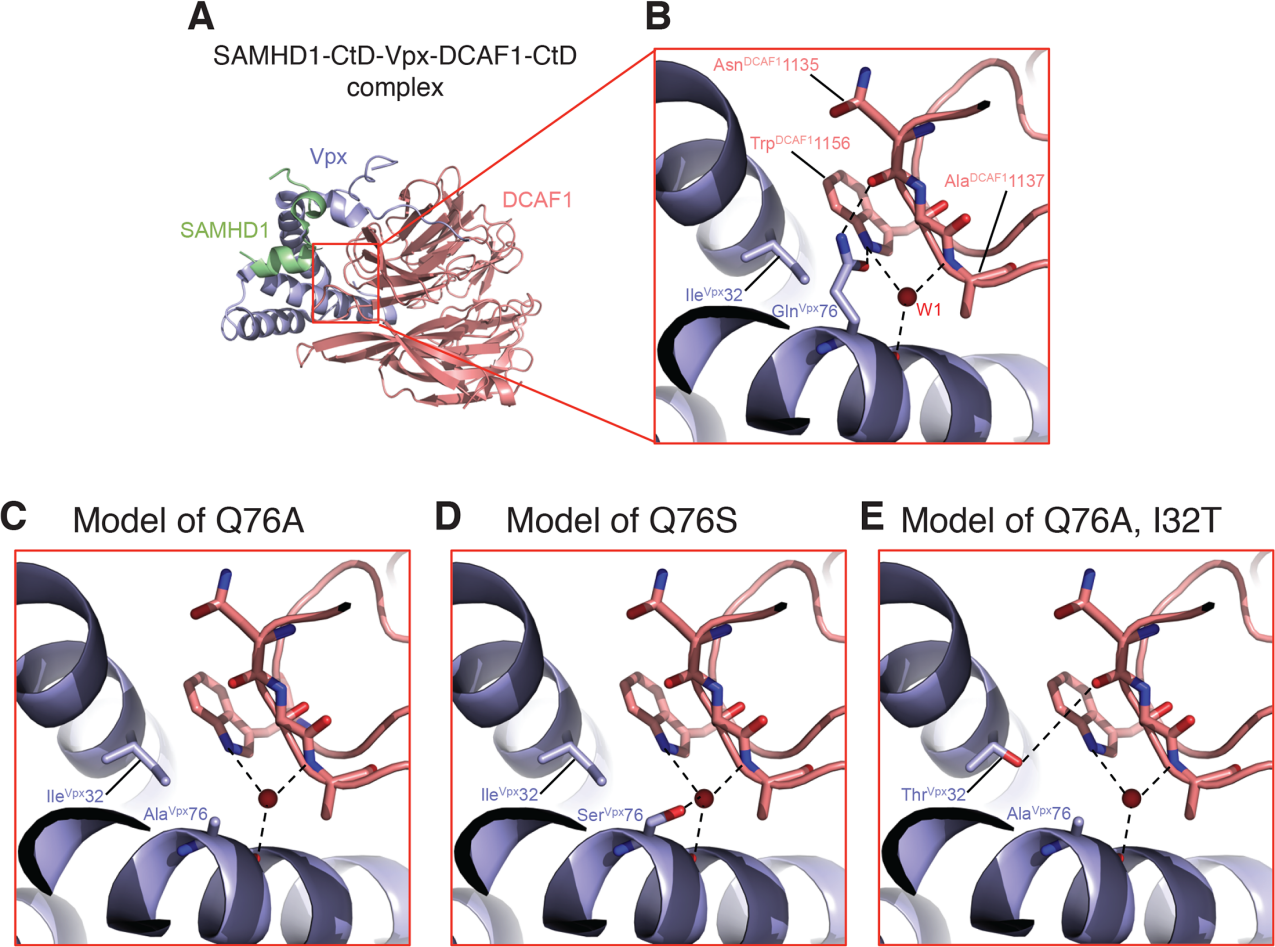
Mapping of Vpx mutations at the Vpx-DCAF1-SAMHD1 interface. (A) Crystal structure (PDB ID 4CC9) of the c-terminal domain of SAMHD1 (green) in complex with WT Vpx (blue) and DCAF1 (salmon). (B) Zoomed-in view displaying the interactions of Gln 76^Vpx^ at the Vpx-DCAF1 interface. Models of the Q76A Vpx mutation (C), the Q76S Vpx substitution (D) in the revertant recovered from macaque K42, and the Q76A/I32T Vpx double substitution in the revertant recovered from macaque JAX4 (E). Hydrogen bonds are indicated by dashed lines and the red spheres denote a crystallographic water molecule observed the structure.

A recent structural study of Vpx and DCAF1 reported that residues Ile32 and Gln76 of Vpx_sm_ both make contact with Trp1156 of the DCAF1 cytoplasmic domain [17]. Vpx residues Ile32 and Gln76 are in close physical proximity within the SAMHD1- DCAF1-Vpx ternary complex with Ile32 making hydrophobic interactions with the aliphatic base of the Gln76 side chain. The carbonyl side chain of Vpx residue Gln76 makes H-bond interactions with DCAF1 Trp1156 and the NH2 side chain of Vpx Gln76 bonds with the main chain carbonyl of DCAF1 Asn1135 (Fig 8A and 8B). In addition, the main chain carbonyl of Vpx Gln76 hydrogen bonds with a water molecule (W1) that mediates extensive contacts between Vpx and DCAF1. Thus Gln76 appears to play a critical role at the Vpx-DCAF1 interface. Modeling the Gln76Ala disabling mutation in Vpx used in this study on the Vpx-DCAF1-SAMHD1 crystal structure places DCAF1 Trp1156 in a predominantly unfavorable hydrophobic environment and creates a potentially destabilizing void at the interface, and also results in loss of interactions mediated by Gln76 in WT (Fig 8C). Substituting the Vpx Ala mutation at position 76 with Ser, as is present in the K42 revertant virus, is predicted to partially fill the cavity created by the original Gln76Ala change and to restore some of the interactions with DCAF1 (Fig 8D). Similarly, the Ile32Thr change, in the presence of the original Vpx Ala mutation, is predicted to restore a hydrogen bond interaction between Vpx and DCAF1 and also stabilizes the interface providing a complementary polar site for DCAF1 Trp1156 to interact with (Fig 8E). Thus by recreating a shared surface to which SAMHD1 binds, Vpx substitutions that are predicted to facilitate interaction with DCAF1 augment SIV replicative capacity *in vivo*.

## Discussion

Our results demonstrate that Vpx is critically important for countering SAMHD1 during SIVmac infections of memory CD4^+^ T lymphocytes both *in vitro* and *in vivo*. SIVs carrying the Q76A Vpx point mutant, which specifically blocks the interaction of Vpx with DCAF1 and the subsequent recruitment of SAMHD1 to the Cullen 4A-ring ubiquitin ligase complex, exhibited an attenuated replication phenotype during infection of ConA activated rhesus macaque PBMC and were deficient in degrading endogenous SAMHD1 in cultured rhesus CD4^+^ T lymphocytes. In a monkey inoculated with WT SIVmac239, levels of endogenous SAMHD1 were markedly reduced in circulating and tissue-associated memory CD4^+^ T cells on day 9 PI of the acute infection, compared to the levels measured in lymphocytes from an uninfected animal. When macaques were inoculated with the T cell tropic SIVmac239 or the macrophage tropic SIVmac316 carrying the Q76A Vpx point mutation, virus acquisition was markedly impaired: multiple IR inoculations were required to establish infections and peak levels of virus production were both delayed and reduced. The maintenance of set point viremia was also severely attenuated in monkeys inoculated with the SIV Vpx point mutation unable to recruit DCAF1. Revertant viruses, which emerged in two recipients of the Vpx point mutant, carried either an A76S change at the original mutation site or an I32T “second site” change, two substitutions located in likely contact points of Vpx with the C-terminal domain of DCAF1. Both SIVmac Vpx revertants exhibited an augmented replication phenotype *in vitro* and *in vivo* and were able to degrade SAMHD1. These results point to a requirement for Vpx to maintain robust SIVmac replication in memory CD4^+^ T cells during all phases of the *in vivo* infection and provide evidence that selective pressure will be exerted in these cells to restore activity occurs if Vpx function is compromised.

Although it has been previously reported that an SIVmne mutant, carrying Vpx changes that prevent binding to DCAF1, exhibited attenuated infectivity in pig-tailed macaques [29], our study is the first to show that expression of functional Vpx during the acute SIV infection causes near complete SAMHD1 depletion in memory CD4^+^ T cells *in vivo*. It must be noted that the Q76A Vpx mutation does not specifically block the interaction of Vpx with SAMHD1, but rather, the binding of Vpx to DCAF1. This may lead to the subsequent recruitment of SAMHD1 to the Cullen 4A-ring ubiquitin ligase complex. On the other hand, the possibility exists that abrogating the binding of Vpx with DCAF1 may allow DCAF interacting cellular partners, other than SAMHD1, to disable SIV replication [30–32].

It is important to point out that 90 to 95% of sorted CD3^+^, CD4^+^, CD8^-^, CD95^+^ memory cells collected from uninfected rhesus macaques are not activated, as measured by Ki67 staining [12]. The median % activation of CD4^+^ T lymphocytes on day 14 post SIVmac239 infection has been reported to be 20% [33]. Thus, a majority of T cells targeted by SIV *in vivo* are not dividing. In this regard, SAMHD1 has been previously reported to suppress virus infection in resting but not in cycling CD4^+^ T cells [5]. The dependence of Vpx restriction on cell activation status was subsequently shown to be due to the phosphorylation state of SAMHD1 [19–21]. This is shown in Fig 3D for SAMHD1, which becomes hyperphosphylated as a consequence of activating rhesus CD4^+^ T cells with the plant lectin ConA. Since infectivity assays required the use of rhesus PBMC, activated with Con A to achieve demonstrable virus replication *in vitro*, the relatively modest attenuated replication phenotype of the SIVmac Q76A Vpx mutants compared to WT SIVmac repeatedly observed in rhesus PBMC (Figs 2 and 7) might simply reflect the increased levels of phosphorylated nonrestrictive SAMHD1 in the stimulated PBMC cultures.

It is now recognized that the gastrointestinal (GI) tract is a major site of HIV-1 and SIV replication and the number of CD4^+^ T cells is markedly reduced during all phases of the virus infection [34,35]. Despite antiretroviral treatment, immune reconstitution in the GI mucosa is variable and incomplete [36,37]. A recent study of 5 monkeys, chronically infected for 2 to 4 years with an SIVmac239 mutant carrying a 101 base pair deletion of the *vpx* gene, reported that virtually no virus infected macrophages were present in lymph node, spleen and colon specimens, collected at the time of their death from immunodeficiency [38]. This result would be consistent with numerous reports showing that Vpx expression is required for efficient replication of SIVmac in myeloid lineage cells. This study had an even more interesting finding. In contrast to animals infected with wt SIVmac239, which experience robust virus replication in the gut-associated lymphoid tissue (GALT) and associated severe sustained depletion of CD4^+^ T cells at this site, the GALT of macaques infected with the SIV Vpx mutant contained very few virus infected CD4^+^ T lymphocytes and had sustained only a minimal loss of this T cell subset. The latter finding suggests that the inability to optimally infect macrophages in the intestinal mucosa may mitigate vigorous virus replication throughout the GALT, thereby significantly altering the typical course of pathogenic lentivirus infections *in vivo*.

At present, it is not clear why HIV-1 neither encodes nor requires an antagonist of SAMHD1 to ensure robust replication in memory CD4^+^ T cells. Perhaps it is due to the reported high activity of its reverse transcriptase compared to that of SIVmac even when intracellular dNTP concentrations are relatively low [39]. Although the mitigating effects of Vpx may be more apparent in cultured cells of the myeloid lineage, which have very high levels of endogenous SAMHD1 (Fig 1) and low levels of dNTPs, our results indicate that endogenous SAMHD1 can potently restrict SIVmac replication in infected rhesus monkeys unless counteracted by Vpx. This need to degrade SAMHD1 during SIV infections of memory CD4^+^ T cells *in vivo* selects for Vpx revertant viruses capable of generating high levels of plasma viremia and inducing immunodeficiency.

## Materials and Methods

### Ethics statement

This study was carried out in strict accordance with the recommendations of the Public Health Services (PHS) Policy of Humane Care and Use of Laboratory Animals. Rhesus macaques (*Macaca mulatta*) were housed in a biosafety level 2 NIAID facility and conducted in accordance with protocols LMM32, approved by the Institutional Animal Care and Use Committees of NIAID/NIH. Appropriate sedatives, anesthetics and analgesics were used during handling and surgical manipulations to ensure minimal pain, suffering, and distress to animals. Furthermore, housing, feeding and environmental enrichment were in accord with recommendations of the Weatherall report. Animals were euthanized in accordance with the recommendations of the panel on Euthanasia of the American Veterinary Medical Association (AVMA) Guidelines for the Euthanasia of Animals (Section 2.3).

### Animal experiments

The macaques used in this study were negative for the MHC class I Mamu-A*01 allele. Phlebotomies, euthanasia and sample collection were performed as previously described [40]. Viral RNA levels in plasma were determined by real-time RT-PCR (ABI Prism 7900HT sequence detection system; Applied Biosystems) as previously reported [40].

### Lymphocyte immunophenotyping and data analysis

EDTA-treated blood samples were stained for flow cytometric analysis as described previously [12,41], using combinations of the following fluorochrome-conjugated MAbs: CD3 (fluorescein isothiocyanate [FITC] or phycoerythrin [PE]), CD4 (PE, peridinin chlorophyll protein-Cy5.5 [PerCP-Cy5.5], or allophycocyanin [APC]), CD8 (PerCP or APC), CD28 (FITC or PE), and CD95 (APC). All antibodies were obtained from BD Biosciences (San Diego, CA), and samples were analyzed by four-color flow cytometry (FACSCalibur; BD Biosciences Immunocytometry Systems). Data analysis was performed using CellQuest Pro (BD Biosciences) and FlowJo (TreeStar, Inc., San Carlos, CA). In this study, naïve CD4^+^ T cells were identified by their CD95^low^ CD28^high^ phenotype, whereas memory CD4^+^ T cells were CD95^high^ CD28^high^ or CD95^high^ CD28^low^ in the CD4^+^ small lymphocyte gate [41].

### SGA of plasma viral RNA

Viral RNA was purified from macaque plasma employing the QIAamp Viral RNA Mini kit (QIAGEN) and immediately converted to cDNA using SuperScript III reverse transcriptase (LifeTechnologies) and a random primer. The newly synthesized single-stranded cDNA was serially diluted and SGA PCR amplification [42] was performed using Platinum Taq High Fidelity polymerase (LifeTechnologies). First round PCR was performed using primers (forward primer: GAAGGGGAGGAATAGGGGATATGAC and reverse primer: CAAAACTGGCAATGGTAGCAACAC) with the following parameters: 1 cycle of 94 C for 2 min, 35 cycles of a denaturing step of 94°C for 15 s, an annealing step of 55°C for 30 s, and an extension step of 68°C for 2 min, followed by a final extension of 68 C for 7 min. Second round PCR was performed using primers (forward primer: CCACTACAGGAAGGAAGCCATTTAG and reverse primer: GCTCCCTCAAGGGTGTCTCCATGTCTATTATA) with the following PCR parameters: 1 cycle of 94 C for 2 min, 45 cycles of a denaturing step of 94°C for 15 s, an annealing step of 55°C for 30 s, and an extension step of 68°C for 2 min, followed by a final extension of 68°C for 7 min.

### Cells

A human embryonic kidney cell line, 293T (HEK-293T, CRL-11268, ATCC, Manassas, VA), was cultured in Dulbecco’s modified minimal essential medium supplemented with 10% heat-inactivated FBS. PM1, and SupT1-R5 cells were cultured in RPMI-1640 supplemented with 10% heat-inactivated FBS. Rhesus monkey PBMCs were prepared, CD8^+^ T cell depleted, and cultured as described previously [43,44]. Rhesus CD4^+^ T lymphocytes were purified by negative selection using a MACS kit ([Miltenyibiotec] for non-human primates). Macaque CD14^+^ cells were purified by CD14 microBeads (Miltenyibiotec) employing a MACS cell isolation system. MDMs were induced from CD14^+^ cells by culturing with 100ng/ml of M-CSF (Peprotech) for 1 week. Adherent alveolar macrophages were isolated from bronchoalveolar lavage (BAL) samples.

### Flow cytometric cell sorting of memory CD4^+^ T lymphocytes from an infected rhesus macaque

Fresh whole blood was diluted 1:1 with phosphate buffered saline (PBS) and layered over Ficoll-Paque Plus (GE Healthcare) and then centrifuged at 2000 rpm for 25 minutes. The PMBC were then removed and washed twice with media, RPMI-1640 supplemented with 10% heat inactivated fetal bovine serum, penicillin, streptomycin and L-glutamine. Whole spleens were digested into single cell suspensions by grinding tissue through a 0.22 μm cell strainer followed by lysis of red blood cells with ACK lysis buffer (Life technologies) and two washes with complete RPMI media. Splenocytes and PBMCs were stained with the live dead exclusion dye Aqua blue (Invitrogen) then with the following mAbs: αCD3-Alexa700 (clone SP34-2, BD Pharmingen), αCD8-Pacific Blue (clone RPA-T8, BD Pharmingen), αCD4-PECy5.5 (clone OKT4, eBioscience), αCD28-ECD (clone 28.2, Beckman Coulter), and αCD95-PECy5 (clone DX2, BD Pharmingen). Memory CD4 T cells were sorted as live, single, lymphocytes expressing CD3, CD4, CD95 without expression of CD8 using a modified FACSAria (BD Immunocytometry Systems). Compensation was performed electronically using capture beads stained singly with the individual mAbs.

### Transfection, infection, and reverse transcriptase assays

Virus stocks were prepared by transfecting 293T cells with SIV molecular clones using Lipofectamine 2000 (LifeTechnologies); culture supernatants were collected 48 h later and stored at −80°C until use. Infectious virus titers were determined by end-point dilution using SupT1-R5 cells. Virion-associated ^32^P-RT activity was measured as described previously [45]. SupT1-R5, and ConA-stimulated rhesus PBMCs (2 × 10^6^ cells in 500 μl) were infected with transfected cell supernatants of indicated viruses (normalized by particle-associated ^32^P-RT activity) by spinoculation [46] for 1 h, and maintained for 12 days. Tissue culture medium was replaced daily and monitored for RT activity.

VSV-G [47] pseudotyped SIVs (WT SIVmac239, SIVmac239 X-del, SIVmac239 X-Q76A) were prepared by transfecting 293-T cells (VSV:SIV plasmid ratio of 1:5) as described earlier. Sorted rhesus memory CD4^+^ T cells, maintained in RPMI-1640 supplemented with 10% heat-inactivated FBS and 20U/ml of IL-2, were spinoculated (1 ml) with pseudovirions present in 293-T cell transfection supernatants. Infected CD4^+^ memory T cells, collected at 24 h post infection, were analyzed for levels of SAMHD1 by immunoblotting.

### Construction of SIVmac Vpx derivatives

Vpx deletion and point mutants were constructed by PCR mutagenesis of SIVmac239 and SIVmac316 genomes using the following primer pairs:

1. SIVmac239 and SIVmac316 X-del, forward primer: GATCCCAGGGAGAGAATCCCACCTGGAAACAG and reverse primer: TTACATCGCTTACTACTTTCAGTGCTAAGTACTGTAGGCTTGG;
2. SIVmac239 and SIVmac316 X-Q76A, forward primer: AGGCTTTATTTATGCATTGCAAGAAAGGCTGTAGATGTCTAGGGGAAG and reverse primer: TTGCTATTAAACACAAGTATCTGTATTTTACATAGCTTGGTGACATCCC
3. SIVmac316 X-Q76S, forward primer: TCAAAGGCTTTATTTATGCATTGCAAGAAAG and reverse primer: TATTAAACACAAGTATCTGTATTTTACATAGCTTGG.
4. The X-Q76A/I32T double mutant was constructed by PCR mutagenesis of SIVmac316 X-Q76A using forward primer: CAAACAGAGAGGCGGTAAACCACCTAC and reverse primer: TCTCCTCTACTGTTCTGTTTAGCCATTCG.

PCR amplifications were performed using Platinum PFX DNA polymerase (LifeTechnologies), with the following PCR parameters: 1 cycle of 94 C for 2 min, 32 cycles of a denaturing step of 94°C for 15 s, an annealing step of 58°C for 30 s, and an extension step of 68°C for 20 min, followed by a final extension of 68 C for 7 min. Following amplification, the PCR product was gel purified, treated with T4 polynucleotide kinase (LifeTechnologies), and then blunt-end ligated to created circular full-length infectious clones.

### Immunoblotting

Cells (2 x 10^6^) were washed with phosphate-buffered saline (PBS) and lysed in 100μl of SDS sample buffer (LifeTechnologies). Whole-cell extracts (15μl [from 3 x 10^5^ cells]) were separated on 10% acrylamide gels (LifeTechnologies) or Phos-Tag gel (Wako Chemicals). Following electrophoresis, the gel was transferred to a PVDF membrane using an iBlot Gel Transfer system (LifeTechnologies)and stained using anti-SAMHD1 (Proteintech), anti-tubulin (Sigma-Aldrich), and anti-GAPDH (Santa Cruz Biotechnology) polyclonal antibodies.

## Supporting Information

S1 Fig Comparison of human and rhesus macaque SAMHD1 amino acid sequences. RNAs were prepared from a mixture of PBMC samples from six different macaques, amplified by RT-RCR, and analyzed by nucleotide sequencing. The deduced macaque SAMHD1 amino acid sequences from 7 amplicons were aligned and compared to that of human SAMHD1. The previously reported Thr 592 residue phosphorylated by cyclin A2/CDK1, which modulates the ability of SAMHD1 to block HIV-1 infection, is indicated in red.
(PDF)

S2 Fig The fraction of memory CD4^+^ T cells in total CD4^+^ T cells declined during WT SIV infections. Rhesus macaques were inoculated intrarectally with 1 x 10^4^ TCID_50_ of SIVmac239 WT or SIVmac239 X-Q76A derivatives (A) or 1 x 10^3^ TCID_50_ of SIVmac316 WT or the SIVmac316X-Q76A derivatives (B). Black curves: WT virus; blue curves: putative revertant Vpx mutants; red curves: non-revertant Vpx mutants.
(PDF)

S3 Fig Alignment of Vpx amino acid sequences amplified from the K42 and JA4X virus swarm stocks. SGA plus sequencing was used to generate *vpx* gene sequences present in the K42 (A) and JA4X (B) virus swarm stocks prepared by cocultivating PBMC from these two infected monkeys with SupT1-R5 cells. The sequences of WT SIVmac Vpx and the starting Q76A Vpx mutant are shown at the top; the animal identifications are indicated on the left. Amino acids highlighted in red represent changes conferring revertant phenotypic changes.
(PDF)
