# Supplementary material for: The Expression of Functional Vpx during Pathogenic SIVmac Infections of Rhesus Macaques Suppresses SAMHD1 in CD4+ Memory T Cells
Source: PLoS Pathog. 2015 May 21;11(5):e1004928. doi: 10.1371/journal.ppat.1004928 (PMC4440783; doi:10.1371/journal.ppat.1004928)
Supplement: S1 Fig — RNAs were prepared from a mixture of PBMC samples from six different macaques, amplified by RT-RCR, and analyzed by nucleotide sequencing. The deduced macaque SAMHD1 amino acid sequences from 7 amplicons were aligned and compared to that of human SAMHD1. The previously reported Thr 592 residue phosphorylated by cyclin A2/CDK1, which modulates the ability of SAMHD1 to block HIV-1 infection, is indicated in red. (PDF) [file ppat.1004928.s001.pdf]

```
                                                                                                                 100
HuSAMHD1     MQRADSEQPSKRPRCDDSPRTPSNTPSAEADWSPGLELHPDYKTWGPEQVCSFLRRGGFEEPVLLKNIRENEITGALLPCLDESRFENLGVSSLGERKKL
RhSAMHD1-1   **Q***D*******F****************CF******************F*******G**A********K************H***************
RhSAMHD1-2   **Q***D*******F****************CF******************F*******G**A********K************H***************
RhSAMHD1-3   **Q***D*******F*********A******CF**************K***F*******G**A********K************H***************
RhSAMHD1-4   **Q***D*******F****************C*******************F*******G**A********K************H***************
RhSAMHD1-5   **Q***D*******F****************C*****F*************F*******G**A********K************H***************
RhSAMHD1-6   **Q***D*******F****************C*******************F*******G**A********K************H***************
RhSAMHD1-7   **Q***D***E***F****************C*******************F*******G**A********K************H***************

                                                                                                                 200
HuSAMHD1     LSYIQRLVQIHVDTMKVINDPIHGHIELHPLLVRIIDTPQFQRLRYIKQLGGGYYVFPGASHNRFEHSLGVGYLAGCLVHALGEKQPELQISERDVLCVQ
RhSAMHD1-1   ******SG***********************************************************************R***************I****
RhSAMHD1-2   ******SG***********************************************************************R***************I****
RhSAMHD1-3   ******SG***********************************************************************R***************I****
RhSAMHD1-4   ******SG***********************************************************************R***************I****
RhSAMHD1-5   ******SG***********************************************************************R***************I****
RhSAMHD1-6   ******SG***********************************************************************R***************I****
RhSAMHD1-7   ******SG***********************************************************************R***************I****

                                                                                                                 300
HuSAMHD1     IAGLCHDLGHGPFSHMFDGRFIPLARPEVKWTHEQGSVMMFEHLINSNGIKPVMEQYGLIPEEDICFIKEQIVGPLESPVEDSLWPYKGRPENKSFLYEI
RhSAMHD1-1   *******************************************************H************************KGV*********R*******
RhSAMHD1-2   *******************************************************H************************KGV*********R*******
RhSAMHD1-3   *******************************************************H************************KGV*********R*******
RhSAMHD1-4   *******************************************************H************************KGV*********R*******
RhSAMHD1-5   *******************************************************H************************KGV*********R*******
RhSAMHD1-6   *******************************************************HH***********************KGV*********R*******
RhSAMHD1-7   *************************************V*****************H************************KGV*********R*******

                                                                                                                 400
HuSAMHD1     VSNKRNGIDVDKWDYFARDCHHLGIQNNFDYKRFIKFARVCEVDNELRICARDKEVGNLYDMFHTRNSLHRRAYQHKVGNIIDTMITDAFLKADDYIEIT
RhSAMHD1-1   ****************************************************************************************************
RhSAMHD1-2   *****************G**********************************************************************************
RhSAMHD1-3   ****************************************************************************************************
RhSAMHD1-4   ****************************************************************************************************
RhSAMHD1-5   ****************************************************************************************************
RhSAMHD1-6   ****************************************************************************************************
RhSAMHD1-7   ****************************************************************************************************

                                                                                                                 500
HuSAMHD1     GAGGKKYRISTAIDDMEAYTKLTDNIFLEILYSTDPKLKDAREILKQIEYRNLFKYVGETQPTGQIKIKREDYESLPKEVASAKPKVLLDVKLKAEDFIV
RhSAMHD1-1   *T*************************************************************E*V***K***********A***RE**E***E******
RhSAMHD1-2   ***************************************************************E*V***K***********A***RE**E***E******
RhSAMHD1-3   ******************************************G********************E*V***K***********A***RE**E***E******
RhSAMHD1-4   ***************************************************************E*V***K***********A***RE**E***E******
RhSAMHD1-5   ***************************************************************E*V***K***********A***RE**E***E******
RhSAMHD1-6   ***************************************************************E*V***K***********A***RE**E***E******
RhSAMHD1-7   ***************************************************************E*V***K***********A***RE**E***E******

                                                                                                   592       600
HuSAMHD1     DVINMDYGMQEKNPIDHVSFYCKTAPNRAIRITKNQVSQLLPEKFAEQLIRVYCKKVDRKSLYAARQYFVQWCADRNFTKPQDGDVIAPLITPQKKEWND
RhSAMHD1-1   ***********N************D**TP*********R*****************M********K*H*******************************Y
RhSAMHD1-2   ***********N************D**TP*********R*****************M********K*H*******************************Y
RhSAMHD1-3   ***********N************D**TP*********R*****************M********K*H*******************************Y
RhSAMHD1-4   ***********N************D**TP*********R*****************M********K*H*******************************Y
RhSAMHD1-5   ***********N************D**TP*********R*****************M********K*H*******************************Y
RhSAMHD1-6   ***********N************D**TP*********R*****************M********K*H*******************************Y
RhSAMHD1-7   ***********N************D**TP*********R*****************M********K*H*******************************Y

                                       626
HuSAMHD1     STSVQNPTRLREASKSRVQLFKDDPM
RhSAMHD1-1   R****S***********L*******K
RhSAMHD1-2   R****S***********LR******K
RhSAMHD1-3   R****S***********L*******K
RhSAMHD1-4   R****S***********L*******K
RhSAMHD1-5   R****S***********L*******K
RhSAMHD1-6   R****S***********L*******K
RhSAMHD1-7   R****S***********L*******K
```

Fig. S1
