# Supplementary figures and images for: The Expression of Functional Vpx during Pathogenic SIVmac Infections of Rhesus Macaques Suppresses SAMHD1 in CD4+ Memory T Cells
Source: PLoS Pathog. 2015 May 21;11(5):e1004928. doi: 10.1371/journal.ppat.1004928 (PMC4440783; doi:10.1371/journal.ppat.1004928)

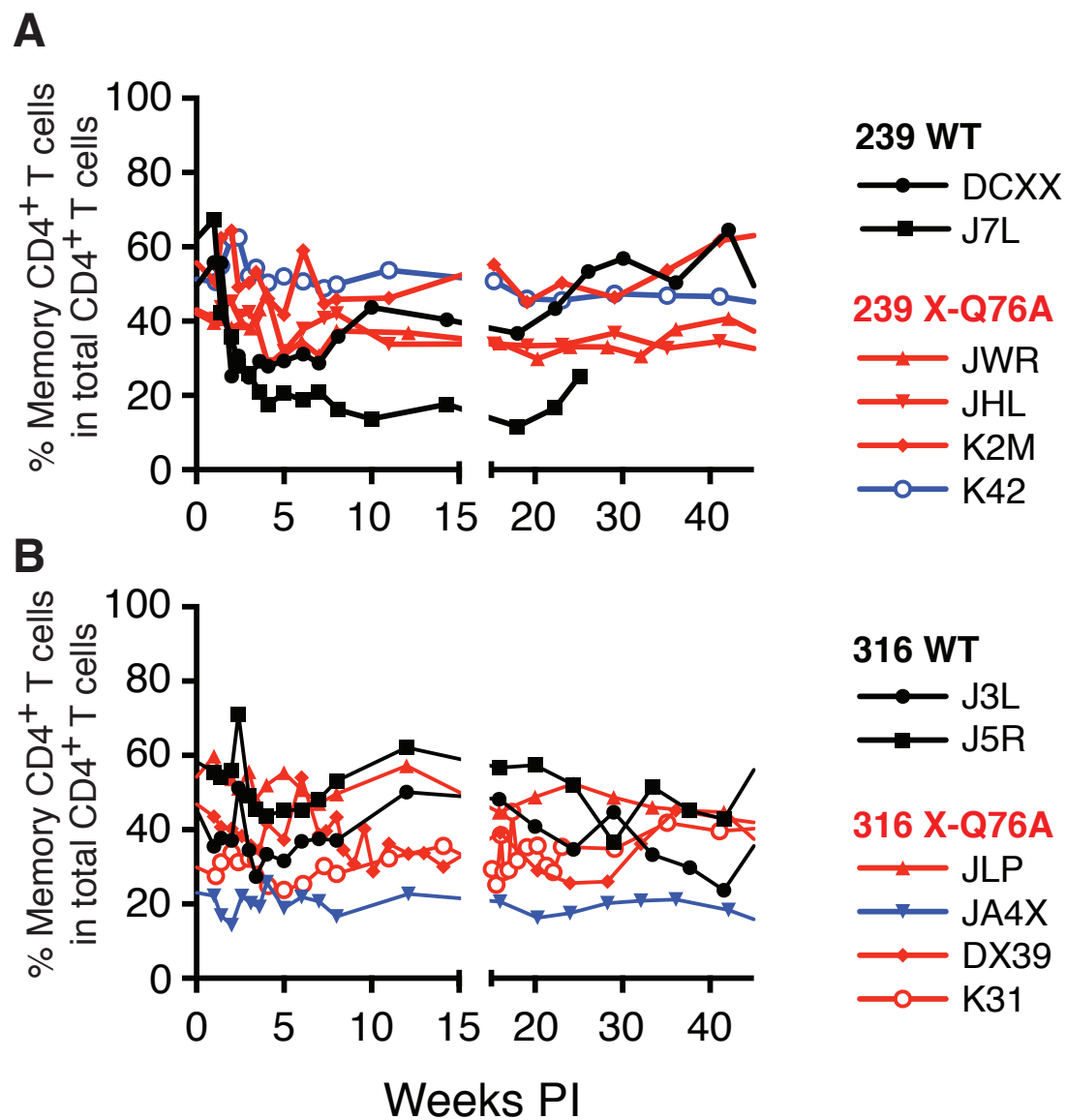

Fig. S2

Supplement: S2 Fig — Rhesus macaques were inoculated intrarectally with 1 x 10^4^ TCID50 of SIVmac239 WT or SIVmac239 X-Q76A derivatives (A) or 1 x 10^3^ TCID50 of SIVmac316 WT or the SIVmac316X-Q76A derivatives (B). Black curves: WT virus; blue curves: putative revertant Vpx mutants; red curves: non-revertant Vpx mutants. (PDF) [file ppat.1004928.s002.pdf]
