# Supplementary material for: The Expression of Functional Vpx during Pathogenic SIVmac Infections of Rhesus Macaques Suppresses SAMHD1 in CD4+ Memory T Cells
Source: PLoS Pathog. 2015 May 21;11(5):e1004928. doi: 10.1371/journal.ppat.1004928 (PMC4440783; doi:10.1371/journal.ppat.1004928)
Supplement: S3 Fig — SGA plus sequencing was used to generate vpx gene sequences present in the K42 (A) and JA4X (B) virus swarm stocks prepared by cocultivating PBMC from these two infected monkeys with SupT1-R5 cells. The sequences of WT SIVmac Vpx and the starting Q76A Vpx mutant are shown at the top; the animal identifications are indicated on the left. Amino acids highlighted in red represent changes conferring revertant phenotypic changes. (PDF) [file ppat.1004928.s003.pdf]

A SIVmac239 Vpx

```
                                                                                        76
WT     MSDPRERIPP GNSGEETIGE AFEWLNRTVE EINREAVNHL PRELIFQVWQ RSWEYWHDEQ GMSPSYVKYR YLCLIQKALF MHCKKGCRCL GEGHGAGGWR PGPPPPPPPG LA
Q76A   MSDPRERIPP GNSGEETIGE AFEWLNRTVE EINREAVNHL PRELIFQVWQ RSWEYWHDEQ GMSPSYVKYR YLCLIAKALF MHCKKGCRCL GEGHGAGGWR PGPPPPPPPG LA
       ********** ********E* ********** ********** *********R ********** ********** *****S**** ********** ********** S********* **
       ********** ********E* ********** ********** ********** ********** ********** *****S**** ********** ********** S********* **
K42    ********** ********E* ********** ********** ********** ********** ********** *****S**** ********** R********* S********* **
swarm  ********** ********E* ********** *V******** ********** ********** ********** *****S**** ********** R********* S********* **
       ********** ********E* ********** ********** ********** ********** ********** *****S**** ********** R********* S********* **
       ********** ********E* ********** ********** ********** ********** ********** *****S**** ********** R********* S********* **
       ********** ********E* ********** ********** ********** ********** ********** *****S**** ********** R********* S********* **
       ********** ********E* ********** ********** ********** ********** ********** *****S**** ********** R********* S********* **
       ********** ********E* ********** ********** ********** ********** ********** *****S**** ********** R********* S********* **
       ********** ********E* ********** ********** ********** ********** ********** ****MS**** ********** R********* S********* **
```

B SIVmac316 Vpx

```
                                       32                                               76
WT     MSDPRERIPP GNSGEETIGE AFEWLNRTVE EINREAVNHL PRELIFQVWQ RSWEYWHDEQ GMSPSYVKYR YLCLIQKALF MHCKKGCRCL GEGHGAGGWR PGPPPPPPPG LA
Q76A   MSDPRERIPP GNSGEETIGE AFEWLNRTVE EINREAVNHL PRELIFQVWQ RSWEYWHDEQ GMSPSYVKYR YLCLIAKALF MHCKKGCRCL GEGHGAGGWR PGPPPPPPPG LA
       ********** ********E* **A******* *T******** ********** ********** ********** ****M***** ********** ********** ********** **
       ********** ********E* **A******* *T******** ********** ********** ********** ****M***** ********** ********** ********** **
       ********** ********E* **A******* *T******** ********** ********** ********** ****M***** ********** ********** ********** **
       ********** ********E* **A******* *T******** ********** ********** ********** ****M***** ********** ********** ********** **
       ********** ********E* **A******* *T******** ********** ********** ********** ****M***** ********** ********** ********** **
JA4X   ********** ********E* **A******* *T******** ********** ********** ********** ****M***** ********** ********** ********** **
swarm  ********** ********E* **A**D**** *T******** ********** ********** ********** ****M***** ********** ********** S********* **
       ********** ********E* **A******* *T******** ********** ********** ********** ****M***** ********** ********** S********* **
       ********** ********E* **A******* *T******** ********** ********** ********** ****M***** ********** *R******** S********* **
       ********** ********E* **A******* *T******** ********** ********** ********** ****M***** ********** ********** S********* **
       ********** ********E* ********** *T******** ********** ********** ********** ****M***** ********** ********** S********* **
       ********** ********E* ********** *T******** ********** ********** ********** ****M***** ********** ********** S********* **
       ********** **N*****E* ********** *T******** ********** ********** ********** ****M***** ********** ********** S********* **
       ********** ********E* ********** *T******** ********** ********** ********** ****M***** ********** ********** ********** **
       ********** ********E* ********** *T******** ********** ********** ********** ********** ********** ********** S********* **
```

Fig. S3
